# Ancient Geographical Gaps and Paleo-Climate Shape the Phylogeography of an Endemic Bird in the Sky Islands of Southern India

**DOI:** 10.1371/journal.pone.0013321

**Published:** 2010-10-13

**Authors:** V. V. Robin, Anindya Sinha, Uma Ramakrishnan

**Affiliations:** 1 National Institute of Advanced Studies, Indian Institute of Science Campus, Bangalore, India; 2 National Centre for Biological Sciences, Tata Institute of Fundamental Research, Bangalore, India; 3 Nature Conservation Foundation, Mysore, India; Midwestern University, United States of America

## Abstract

**Background:**

Sky islands, formed by the highest reaches of mountain tracts physically isolated from one another, represent one of the biodiversity-rich regions of the world. Comparative studies of geographically isolated populations on such islands can provide valuable insights into the biogeography and evolution of species on these islands. The Western Ghats mountains of southern India form a sky island system, where the relationship between the island structure and the evolution of its species remains virtually unknown despite a few population genetic studies.

**Methods and Principal Findings:**

We investigated how ancient geographic gaps and glacial cycles have partitioned genetic variation in modern populations of a threatened endemic bird, the White-bellied Shortwing *Brachypteryx major*, across the montane *Shola* forests on these islands and also inferred its evolutionary history. We used Bayesian and maximum likelihood-based phylogenetic and population-genetic analyses on data from three mitochondrial markers and one nuclear marker (totally 2594 bp) obtained from 33 White-bellied Shortwing individuals across five islands. Genetic differentiation between populations of the species correlated with the locations of deep valleys in the Western Ghats but not with geographical distance between these populations. All populations revealed demographic histories consistent with population founding and expansion during the Last Glacial Maximum. Given the level of genetic differentiation north and south of the Palghat Gap, we suggest that these populations be considered two different taxonomic species.

**Conclusions and Significance:**

Our results show that the physiography and paleo-climate of this region historically resulted in multiple glacial refugia that may have subsequently driven the evolutionary history and current population structure of this bird. The first avian genetic study from this biodiversity hotspot, our results provide insights into processes that may have impacted the speciation and evolution of the endemic fauna of this region.

## Introduction

Geographical isolation is one of the major drivers of speciation as exemplified by species on islands. Although most research on biogeographic isolation has been associated with oceanic islands, recently continental islands or “sky-islands” have received increasing attention [1], [2]. Sky islands are a continental terrain of valleys and mountains where the mountains, like oceanic islands, may act as isolated cradles of evolution [1], [2]. Sky islands, however, are different from oceanic islands in that intervening valleys may act as barriers or may turn into bridges for dispersal depending on the ecology of the species and the conditions in the valley. Such a dynamic system, where both isolation and connectedness are possible has been shown to impact species' distributions and population structure [3]. The interplay between such processes result in these islands harbouring high endemism; 96 species of vertebrates in the Eastern Arc [4], over 200 species in the New Guinean montane habitats [5] and 22–100% of various taxa in the Mexican sky islands [6]. This makes them important regions to target conservation efforts.

The Western Ghats mountains of southern India are identified as a global biodiversity hotspot [7] and a “globally outstanding ecoregion” [2]. The mountains are a 1600-km long chain that forms one of the most isolated sky-island systems in the world [1]. Like other sky island systems of the world, the Western Ghats sky islands also host a large number of endemic species. A third of all Indian plant species, half the reptiles and 75% of the amphibians are in the high elevation montane forests [8]. Almost no studies (but see [9]) have examined the biogeographic affinities of these species, and very little is known about the relationship between species distribution patterns and major geographical barriers in the Western Ghats.

**Figure 1:**
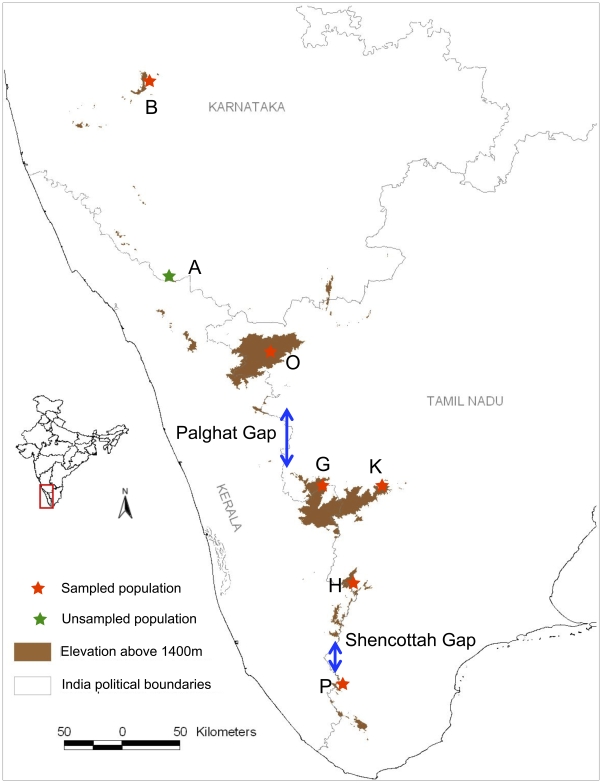
Sky islands and geographical gaps of Western Ghats in southern India and sampling locations for White-bellied Shortwing. Population codes: B – Bababudan, A – Brahmagiri, O – Ooty, G – Grasshills, K – Kodiakanal, G+K – Anamalai, H – High-Wavies, P – Peppara.

Unlike the well-studied Madrean and Eastern-Arc sky island systems [10], [11], [12] there exists no information on the influence of topography on the phylogeography of a Western Ghats species. The Western Ghats has three major geographical breaks, the youngest (65–80 Million years ago (Mya)) and northernmost (16°N) Goa Gap [13], [14] and the two older (500 Mya) gaps (Fig 1), the Palghat Gap (widest, 40 km at 11°N) and the Shencottah Gap (narrowest, 7.5 km at 9°N) [15], [16]. There have been very few studies examining the impact of these gaps on the distribution [17], [18] or population genetic structure [19]–[21] of different species in the Western Ghats.

Unlike continuously distributed large mammals (e.g. Asian elephants), animals that inhabit montane sky islands could have different population histories. For example, the sky islands could have been colonised multiple times from a common source population. Alternatively, a single sky island could have been colonised, followed by subsequent dispersal to other islands through successive, serial founder events. The latter, also known as the ‘stepping stone’ model is one where genetic data would reveal a signature of isolation by distance [22]. In such a scenario, the populations closest to each other would be similar compared to populations that are distant [3], [23]. Given their relatively linear structure, the Western Ghats and their sky-islands provide an ideal system to test whether genetic variation is distributed in an isolation-by-distance pattern.

The evolutionary history of a species may also be affected by the conditions prevailing at different periods of time. Because they impact species distributions, global climatic fluctuations (e.g. the glacial cycles of the Quaternary period) have had a profound impact on the genetic structure and evolution of species and communities [24]–[26]. Glacial cycles may result in fragmentation of a widespread ancestral species into single or multiple refugia [27], [28], resulting in genetic divergence among small isolated populations, especially in sky islands, hence driving speciation (reviewed in [29]). Very little is known about the paleo-environment of south Asia [30] particularly the impacts of quaternary climatic transitions on the flora and fauna of the Indian subcontinent (but see [30]–[32]).

In order to investigate the impacts of biogeographic barriers, habitat structure and the possible impacts of Quaternary climate transitions on sky-island species in the Western Ghats, we chose the White-bellied Shortwing *Brachypteryx major* as our study species because it is a small (<25 cm, <25 g) passerine bird that is restricted only to the high-elevation *Shola* forests (above 1400-m) on the sky islands of the Western Ghats south of 13.5°N [33], [34]. The species occurs in discrete populations that are separated by the Palghat (40 km wide) and the Shencottah (7 km wide) Gaps as also by great geographical distances (>80 km) (Figure 1).

It should be noted that the species has also been referred to as two separate species, the Nilgiri Blue Robin *Myiomela major* and White-bellied Blue Robin *Myiomela albiventris* [18] but the genus has also been referred to as *Callene* by the same author [35]. As this proposal was not substantiated by any rigorous taxonomic data and as the taxonomic position of the taxon remains unclear, debatable and subject to further research, we focus this study on the evolutionary history of this bird and adopt the commonly accepted name White-bellied Shortwing for both sub-species [17], [36].

In this paper, we examine the population structure of the White-bellied Shortwing to investigate (a) whether geographical gaps affect its population structure and (b) whether the population genetic structure of the species reflects the island structure of the sky islands of the Western Ghats. Further, we investigate the evolutionary history of this bird to determine whether (a) dispersal between sky islands for the Shortwing follows a stepping stone model or if the population is the product of multiple vicariance events and (b) the timing of these dispersal events correlates with global climatic events.

## Results

### Geographical gaps and the Shortwing

**Figure 2:**
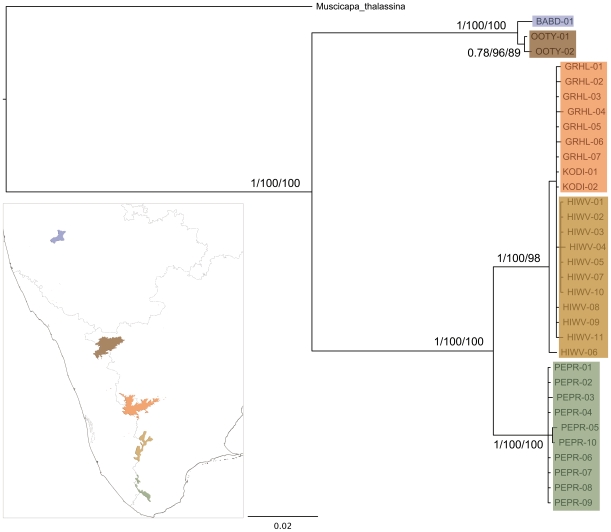
Phylogenetic tree (Bayesian) of White-bellied Shortwing on sky-islands of southern India based 1756 bp on cytochrome-b and cytochrome oxidase-1 marker. Branch support values indicate Bayesian, Neighbour-Joining bootstrap support and Maximum Likelihood bootstrap support and values; tree topology was determined by Bayesian analysis. The samples from a region are coloured by ‘island’ colour in the map.

We found that the geographical gaps in the Western Ghats significantly affected the population structure of the Shortwing. The deepest phylogenetic division corresponds to the Palghat Gap as revealed by different methods including Bayesian analysis (Figure 2, Supplementary Figures: S1, S2, S3). The two population groups on either side of the Gap are reciprocally monophyletic. The second deepest split correlates with the Shencottah Gap (Figure 2, Supplementary Figures: S1, S2, S3), while populations within these Gaps were relatively less structured. The nuclear marker G3PDH, however, did not show any resolution between these clades (Supplementary Figure: S4).

**Figure 3:**
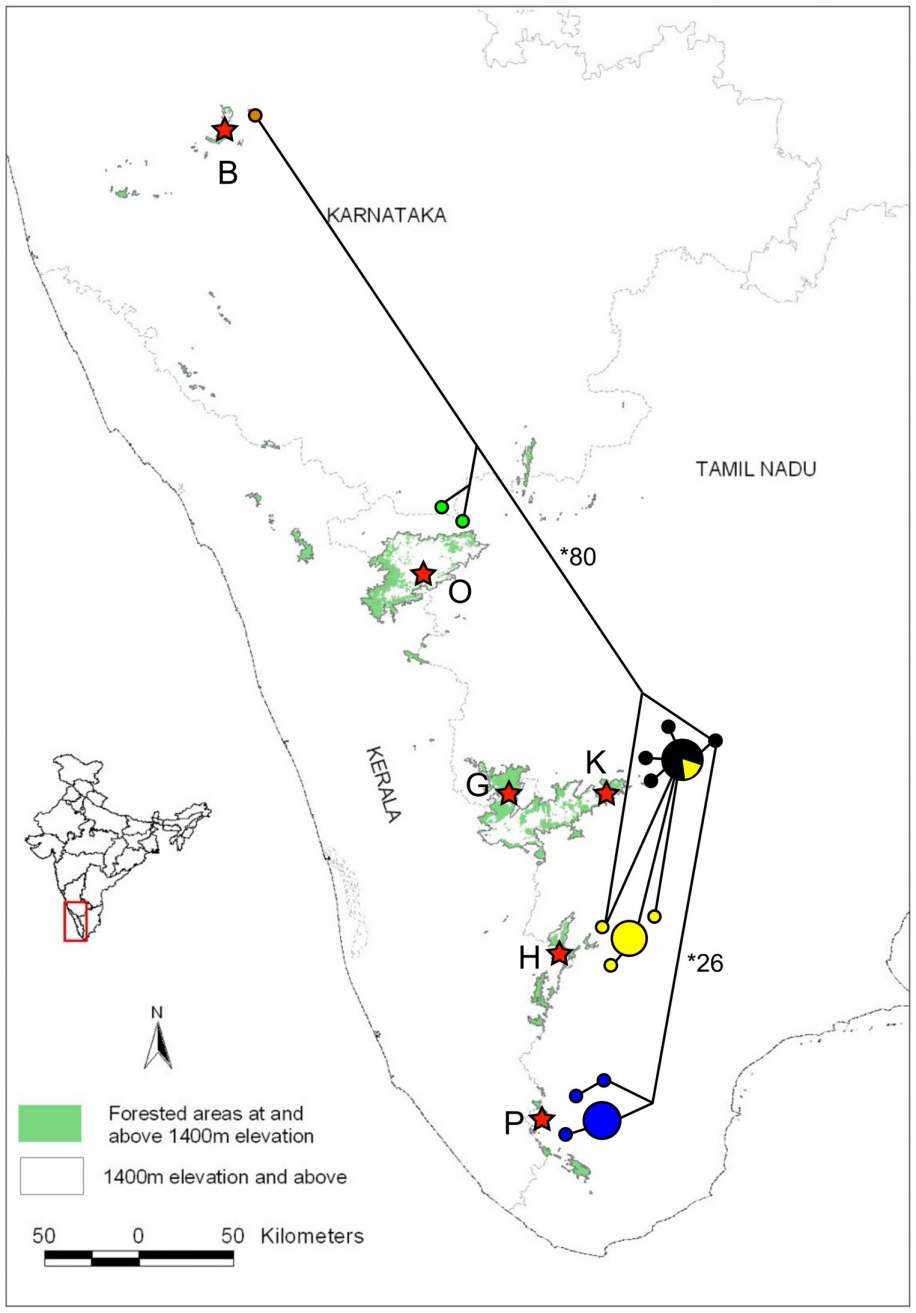
Haplotype network of White-bellied Shortwing on sky-islands of southern India based on 1067 bp of cytochrome-b marker using the median-joining (MJ) network algorithm. Population codes: B – Bababudan, O – Ooty, (G - Grasshills + K- Kodaikanal) – Anamalai, and H –High-Wavies, P – Peppara.

The genetic distance (Kimura-2 parameter, Table 1) within the population groups revealed that most groups from different regions had very low within-group variation for virtually all markers except D-loop. The highest between-group distances (about 9% and above) separated the Bababudan and Ooty populations north of the Palghat Gap from the Anamalai (comprised of Grasshills and Kodaikanal), High-Wavies and Peppara populations to the south of it. The next highest difference was between populations across the Shencottah Gap: the Anamalai and High-Wavies populations exhibited more than 2% distance from the Peppara population (Table 1). These results were consistent across most of the markers tested (Table 1). The haplotype network (Figure 3) revealed that populations on either side of the Palghat Gap showed the highest number of nucleotide differences (80) and shared no haplotypes. The populations on either side of the Shencottah Gap showed the next highest number of differences (26) and also shared no haplotypes.

**Table 1:** Kimura-2-parameter genetic distance and F_ST_ of sampled White-bellied Shortwing populations in the Western Ghats sky islands.

	K2P+ distance						F_ST_				
	Within Groups	Between Groups					BaBd	Ooty	Grhi	Kodi	HiWv
		BaBd	Ooty	Grhl	Kodi	Hiwv					
*Cox-1*											
**Ooty**	0.00	0.56	-	-	-	-	1.000	-	-	-	-
**GrHl**	0.08	9.21	9.22	-	-	-	0.992*	0.991	-	-	-
**Kodi**	0.00	9.17	9.18	0.04	-	-	1.000	1.000	0.000	-	-
**HiWv**	0.05	9.17	9.18	0.06	0.02	-	0.995*	0.994	0.013	0.000	-
**Pepr**	0.00	8.90	8.90	2.61	2.58	2.58	1.000*	1.000	0.987*	1.000*	0.989*
*Cyt-b*											
**Ooty**	0.56	0.57	-	-	-	-	0.000	-	-	-	-
**GrHl**	0.08	8.46	8.62	-	-	-	0.990	0.981*	-	-	-
**Kodi**	0.09	8.48	8.64	0.00	-	-	0.989	0.959	0.032	-	-
**HiWv**	0.13	8.50	8.65	0.14	0.15	-	0.983*	0.978*	0.233	0.173	-
**Pepr**	0.07	8.59	8.75	27.2	2.75	2.80	0.991*	0.985*	0.972*	0.973*	0.962*
*D-loop*											
**Ooty**	0.00	0.25	-	-	-	-	1.000	-	-	-	-
**GrHl**	0.48	12.55	12.86	-	-	-	0.959	0.966*	-	-	-
**Kodi**	1.52	12.32	12.63	0.94	-	-	0.870	0.936	0.194	-	-
**HiWv**	1.15	12.46	12.77	0.99	1.36	-	0.902	0.915*	0.154*	0.869	-
**Pepr**	1.66	13.45	13.76	4.51	4.43	4.23	0.869	0.887*	0.738*	0.625*	0.666*
*MtDNA*											
**Ooty**	0.28	0.51	-	-	-	-	0.454	-	-	-	-
**GrHl**	0.15	9.43	9.57	-	-	-	0.983	0.981*	-	-	-
**Kodi**	0.32	9.40	9.53	0.22	-	-	0.964	0.967	0.117	-	-
**HiWv**	0.29	9.56	9.57	0.23	0.32	-	0.967	0.968*	0.164*	0.927	-
**Pepr**	0.33	9.68	9.69	3.00	2.99	2.98	0.963	0.964*	0.913*	0.888*	0.895*
*G3PDH*											
**Ooty**	0.000	0.000	-	-	-	-	0.000	-	-	-	-
**GrHl**	0.001	0.004	0.004	-	-	-	0.630	0.700*	-	-	-
**Kodi**	0.000	0.003	0.003	0.001	-	-	1.000	1.000	0.000	-	-
**HiWv**	0.000	0.003	0.003	0.001	0.000	-	1.000	1.000*	0.254	0.000	-
**Pepr**	0.001	0.003	0.003	0.001	0.001	0.001	0.704	0.747*	0.000	0.000	0.124

*significant (p<0.05),

+ Kimura-2-Parameter model;

Population codes: BaBd – Bababudan, Grhl- Grasshills, Kodi - Kodiakanal, HiWv – High-Wavies, Pepr – Peppara; Marker codes: cyt *b* - cytochrome *b* , cox - cytochrome *c* oxidase subunit I, D-loop - Control Region, G3PDH - glyceraldehyde-3-phosphate dehydrogenase intron.

We also found that geographical gaps play a more important role in structuring the genetic variation in the species than does geographical distance, with poor support for an isolation-by-distance model. Both genetic distance (F_ST_, Mt-DNA) and geographical gaps (Z = -0.1861, r = 0.858, p<0.05) correlated with geographical distance (Z = -2.7084, r = 0.5923, p<0.05) but partial correlations revealed that genetic distance was significantly correlated to gaps (r = 0.8360, p<0.05) but not to geographical distance (r = 0.5088, p>0.05) when the third factor was controlled for.

### Different species

The barcoding marker, COI, showed a genetic distance of 8.9 to 9.2% between the populations north and south of the Palghat Gap (Ooty vs Anamalai, High-Wavies, Peppara). This value is higher than that recognized as the threshold value of 7.9% between an average pair of avian species [37].

### Sky Islands and population genetic structure

We found that the population genetic structure of the Shortwing did not reflect the island structure of the Western Ghats. AMOVA revealed that a model with three major populations had the most support when individuals from five islands were sampled (Table 2). The Bababudan and Ooty populations, north of the Palghat gap, form the first population group while the Grasshills-Kodaikanal-High-Wavies populations, south of the Gap, and the Peppara population, south of the Shencottah Gap, formed the other two. The Grasshills-Kodaikanal and the High-Wavies populations, within the second group, which we denote as the ‘central-island complex’, comprised two separate geographical islands. Samples from these two islands, Grasshills-Kodaikanal (comprising the Anamalais group) and the High-Wavies were also found to be part of the same phylogenetic clade with no resolution between these regions, as revealed by different analyses conducted using cytochrome *b* (cyt *b*), cytochrome *c* oxidase subunit I (COI) and Control Region (D-loop), the nuclear marker glyceraldehyde-3-phosphate dehydrogenase intron (G3PDH) and the combined mitochondrial DNA (cyt *b* and COI) sequences (Figure 2, Supplementary Figures: S1, S2, S3, S4). Grasshills and Kodaikanal on the same island were found to be genetically similar (genetic distance, haplotype network; Figure 3).

**Table 2:** Population structure of White-bellied Shortwing on sky-islands of southern India based on Analysis of Molecular Variation (AMOVA).

Region	% Variation		
	Between groups	Among population, within group	Within population
Cox-1	99.13	0.05	0.82
Cyt-b	96.55	0.81	2.64
D-loop	86.68	1.09	12.23
MtDNA	93.04	0.75	6.22
G3PDH	51.00	5.18	43.83

Footnote: The population structure specified in this analysis – populations and individuals considered together as groups are enclosed in brackets - (Ooty+Bababudan), (Grasshills+Kodaikanal+High-Wavies), (Peppara); Marker codes: cyt *b* - cytochrome *b* , cox-1 - cytochrome *c* oxidase subunit I, D-loop - Control Region, G3PDH - glyceraldehyde-3-phosphate dehydrogenase intron.

For all investigated markers (except D-loop and G3PDH) the three-population-model showed the maximum variance between groups and the lowest among populations within each group (Table 2). These results were supported by analyses, which showed that the highest pair-wise F_ST_ values were for populations across the Palghat Gap and the next for those across the Shencottah Gap. The Bababudan population showed a high level of differentiation compared to all the other populations for most regions but this was not statistically significant, possibly due to low sample size. The haplotype network also showed that the Grasshills-Kodaikanal-High-Wavies populations, which lay between the two Gaps, shared the maximum number of haplotypes and showed the least number of mutations (Figure 3). The Bababudan, Ooty and Peppara populations formed separate phylogenetic clades and they were all on different islands.

### Evolutionary history of the Shortwing

**Figure 4:**
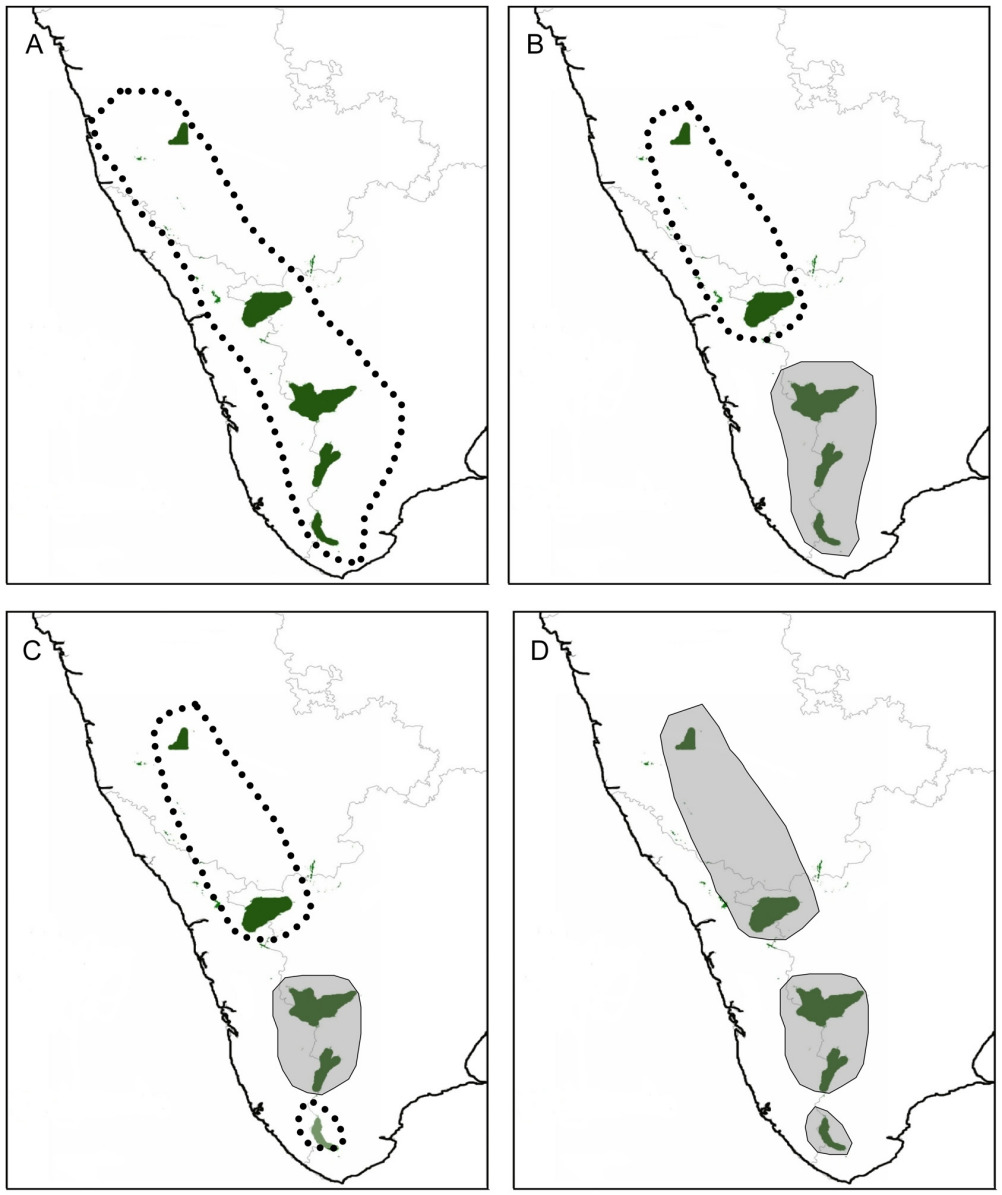
Hypothesized evolutionary history of White-bellied Shortwing on sky-islands of southern India based on Bayesian molecular dating with cytochrome-b marker. Dotted line indicates possible distribution: A) 5 Mya, B) 1.5 Mya, C) 35 Kya, and D) 20 Kya. Population codes: B – Bababudan, O – Ooty, A – Anamalai, H – High-Wavies, P – Peppara.

Divergence estimates, based on cyt *b* sequence analyses, revealed that the time to most recent common ancestor (TMRCA) for all populations of White-bellied Shortwing was 4.909±0.003 Mya. The southern population is the oldest one with a TMRCA of 1.566±0.001 Mya. Subsequently, the Anamalai group was colonised about 0.342±0.001 Mya, Peppara about 0.224±0.001 Mya and the northern populations of Bababudan and Ooty about 0.255±0.0004 Mya (Figure 4, A-D). The time of expansion of the Peppara population, based on the Tau statistic of the mismatch distribution, though based on a limited sample size, occurred 23,000 years ago while the Anamalai group-High-Wavies population expanded around 35,000 years ago, closely corroborating the dates estimated by the Bayesian analysis of the cyt *b* sequence. These estimated dates of divergence of the study populations clearly show that the stepping stone model of dispersal cannot explain the evolutionary history of the Shortwing.

## Discussion

### Role of geographic gaps

We proposed to examine the effect of geographical gaps on the population genetic structure of the Shortwing. We found that the magnitude of genetic difference between the populations of Shortwings corresponded to the size of the geographical gap and this was supported by multiple analyses: genetic distance, F_ST_, phylogenetic trees and network analysis. The highest genetic difference is across the widest geographical gap, the Palghat Gap while the next highest genetic difference was in populations across the second widest gap, the Shencottah Gap. The isolation by distance tests also confirmed that geographical gaps played a relatively more important role than geographical distance in the genetic differentiation of the Shortwing. The Shortwing populations on either side of the Palghat Gap also showed difference in plumage colour. The level of mitochondrial DNA divergence seen across the two Indian Gaps reflects the kind of divergence seen in other systems like the sky island species of the Eastern Arc mountains (12 to 1.5%) across a 110-km lowland gap [38]. In the specific case of the Palghat Gap in the Western Ghats, it is known to be a barrier for gene flow for different organisms (elephants [21], a caecilian [39], and some plants [20]). Here we showed for the first time that the Palghat gap functions as a genetic barrier for any bird species.

In contrast, the Shencottah Gap has not been identified as a geographical barrier for the distribution of species (except Grey-breasted Laughing Thrush [40]). Here, for the first time, our results highlight the critical role this gap may have played in the evolution of species occurring in the Western Ghats, although this needs to be tested with other species. Unlike Shortwing populations on either side of the Palghat gap that differ in plumage, there were no differences in the plumage of individuals on either side of the Shencottah gap, reiterating that genetic differentiation could be morphologically cryptic. There have been other studies that have shown that morphologically similar species may show cryptic genetic differentiation [41]. As with Shortwings, this gap may also play an important and so far completely undetected role in the population structure of several species.

### Different species

Based on plumage and morphological differences between populations of Shortwings across the Palghat Ghat, there has been some debate [18], [36] regarding the validity of two sub-species - *Brachypteryx major major* and *B. m. albiventris*. Our data clearly show that the genetic distance between these populations is higher than that accepted for two avian populations to be considered as two different species based on the genetic analysis of the bar-coding region (COI). We find that the two populations are reciprocally monophyletic and hence, can be classified as two different species. We propose that based on the common names followed in Ali and Ripley [17] the northern species be called the Rufous-bellied Shortwing *Brachypteryx major and* the southern species the White-bellied Shortwing *Brachypteryx albiverntris*.

### Population structure and island structure

The results from the current data set reveal that the Shortwings have a complex population structure. In places where breaks between islands correspond to geographical gaps there is a correlation between physiography and genetic structure. In other areas, however, the island structure does not correlate with genetic structure. The central-island complex of Grasshills, Kodaikanal and High-Wavies form a single large population (phylogenetic tree topology, haplotype Network, AMOVA) although they were on two different islands. These samples showed very low genetic distance across all genetic markers despite a comparable high sample size. However, the structure within this region, especially Grasshills and Kodaikanal, is not well resolved. As different genetic markers revealed different sub-structures, we cannot derive strong inferences about the structure within this region with the present data. The observed lack of structure could be due to recent divergence or ongoing gene flow between these populations. Data from faster-mutating regions like microsatellites might clarify our understanding of within-island population structure. For example, although Kodaikanal and Grasshills are on the same island, they are currently not connected by forests (Figure 3). Genetic data reinforce that they are still part of the same population, denoted here as Anamalai population. High-Wavies, a separate island, is nearly equidistant to the other islands, Peppara and Anamalai; however, it is closer to Anamalai in genetic distance, number of shared haplotypes and F_ST_ values, thus forming a single population.

**Figure 5:**
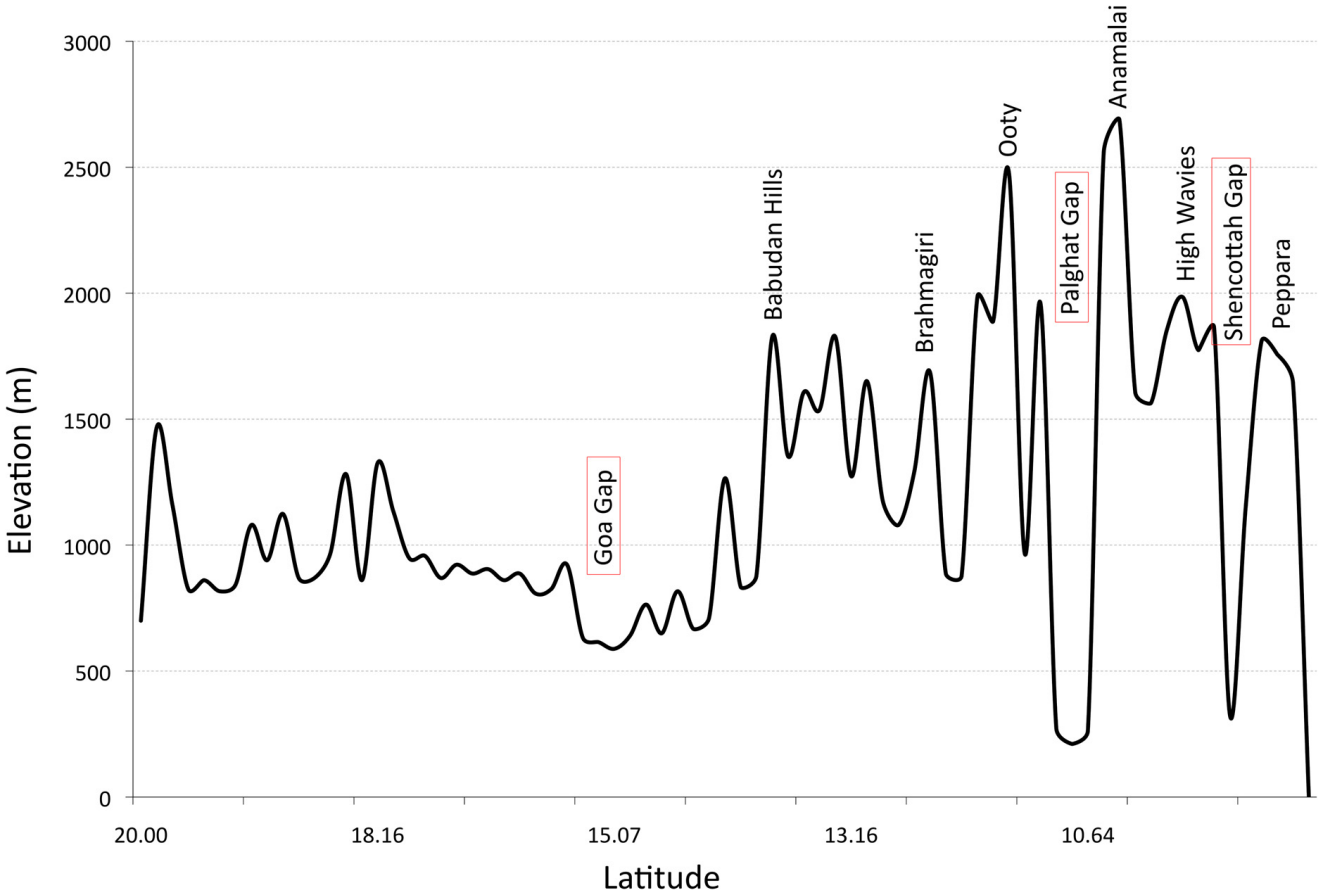
Elevational profile of the Western Ghats showing the geographical gaps and sky islands. Adapted from Ramesh *et al.* [39].

In order to gain a better understanding of the impacts of physiography on patterns of genetic structure and to explore why certain populations stayed connected, we mapped the elevational profile of the Western Ghats [42]. Figure 5 reveals that the geographical gaps corresponding to the population structure breaks go down in elevation to about 300 m while the ‘islands’ of the High-Wavies and Anamalai that do not show a break in population structure are connected by a 1000-m elevation region. Considering that Shortwings are occasionally reported from areas between 1000 m to 1500 m (although the high density Shortwing habitats are above 1500 m [33], [34]), it is possible that Shortwings may be using this elevation for dispersal. Bababudan and Ooty populations on different northern islands were found to be separated even based on the slower mutating nuclear introns, indicating that perhaps this may be an old split that would be further resolved with higher sample sizes from this region. These two islands are also not connected by regions above 1000 m and may hence be a dispersal barrier. We propose that the depth or minimum elevation between islands may be driving the population structure of this species. This also provides us with a hypothesis that can be conclusively investigated by sampling the Brahmagiris, the only unsampled island that is connected to the Ooty island by elevations lower than 1000 m though they are geographically very close.

### Evolutionary history

If the Shortwing dispersed between different sky islands following the stepping stone model, we would find the oldest population on one end of the linear island system with younger populations arising from it. However, the evolutionary history of the Shortwing does not follow the stepping stone model. The evolutionary history seems to be more complex, again implicating a strong role of geography and climate in this region. The split between the north and south species at the Palghat Gap is the oldest, about 5 Mya. Primary rainforest songbirds in Africa have been known to show a Miocene origin [43] and this has been subject to fairly detailed review by Fjeldsa and Brown [38]. The two species of Shortwings have evolved very close to this time frame of origin of African rainforest songbirds. Since no closely related species of the Shortwing inhabit the Western Ghats, it is difficult to propose hypotheses regarding the past distribution or original habitat of this species. The southern population of the Shortwing (Anamalai, High-Wavies and Peppara) diverged about 2 Mya from the ancestral population and this divergence is nearly at the time that other African montane forest birds like the Starred Robin *Pogonocichla stellata* [44] and other montane highland species that diversified at Plio-pleistocene [38]. This event also seems to have occurred at the same time as some Plio-pleistocene speciation events in Africa that have also been proposed to be driven by paleo-climate [45], [46].

The most interesting aspect of the evolutionary history of the species is that populations north and south of the gaps show deep and old splits, whereas the present populations within the three clades have diverged very recently, in the Pleistocene, correlating with the Last Glacial Maximum. We also detected signatures of rapid population expansion for all these young populations based on mismatch distributions. Hence, we propose that major species divergence occurred very early during the Pliocene glacial cycle events along the major geographical gaps. It is likely that these ancestral populations underwent a bottleneck or population crash forming multiple glacial refugia on these islands. The current populations are clearly re-colonised and are expanding from a recent founder event, with their dispersal being continually restricted by the geographical gaps. Unlike African montane regions, where some peaks have recent volcanic origin, causing much of the speciation, there is no known volcanic activity in the Western Ghats in the recent times and much of this region has rocks that are of Archean (2400 Mya) origin. The origin of the geographical gaps (>500 Mya) also predates the origin of passerines in the Tertiary [47]. Habitat reconstruction from recent studies based on palynology and oxygen isotopes have shown that, between 35 to 25 thousand years ago (Kya), the area covered by Anamalai and High-Wavies was inhabited by tropical montane forests ([30], [31], reviewed in [32]). This habitat might hence have served as ‘Pleistocene refugia’ when surrounding habitat was dry like on other sky islands ([38], but see [48]). The unique geography and change in climate conditions would have allowed tropical forests to persist on these islands through the glacial periods as seen in the Eastern Arc mountains of Africa (reviewed in [49]). The timing of these events correlate with population trends of Shortwings as revealed by this study.

Based on our results, for the first time, we can make predictions regarding phylogeographic patterns for species in the Western Ghats mountains of the Indian subcontinent. This is significant not only for the Western Ghats, but for peninsular India as a whole, because this region lacks a phylogeographic paradigm. Our results suggest that biogeographic divides might affect relatively small, endemic Western Ghats species that occupy the sky islands. On the other hand, more vagile, larger species that live at lower elevations are less likely to be affected by these divides. More generally, the extent to which these divides will shape population structure will depend on the elevational range the species occupies and its dispersal ability. The lack of stepping stone based dispersal in the evolutionary history of the Shortwing might be a general pattern shown by other species, or might be limited to species of certain life histories and ecologies. Future studies that use a comparative phylogeographic approach, examining multiple species with different body sizes, ecological niches and life histories would be the best approach to understand shared aspects of evolutionary history and ecological response between species.

In conclusion, our study reveals that ancient geographical gaps clearly form barriers for dispersal and gene flow and have caused deep divergences in a montane species, while habitat and climate refugia have influenced recent population history. The gaps and physiography, hence, play a background role that is more proximally affected by habitat and climate changes. For the first time, we demonstrate the impacts of both biogeographic barriers and recent quaternary climatic events on a bird species in the Western Ghats mountain chain in the Indian subcontinent. Our results have important implications for the White-bellied Shortwing, and also provide, much-needed phylogenetic predictions and hypotheses that could be tested in future studies not only in this biodiversity-rich, yet under-studied, region of the world but also in other sky island systems across the world.

## Materials and Methods

### Field sampling

Blood samples were collected from 33 individuals of the Shortwing from altitudes above 1400 m on the five sky islands namely Bababudan Hills, Nilgiri Hills, Anamalai Hills, Cardamom Hills and Agasthyamalai Hills (Figure 1). Sampling was conducted at six different sites Bababudan Hills - Karnataka (n = 1), Ooty – Nilgiri Hills (n = 2), Grasshills – Anamalai Hills (n = 7), Kodaikanal – Anamalai/Palni Hills (n = 2), High-Wavies – Cardamom Hills (n = 11) and Peppara – Agasthyamalai Hills (n = 10) on these islands (Figure 1) covering nearly all known populations of the species. Five to 15 mist nets (12.5 m×2 m, mesh size 15 mm) were used with the playback of songs of breeding males to attract Shortwings into mist nets. Blood collection followed Sutherland *et al.* [50]. We collected 10–20 µl blood from the ulnar vein using a heparinised micro-haematocrit capillary tube and stored in blood lysis buffer at room temperature in the field and later at −20°C in the laboratory. Although research on wild species in their natural habitats in India generally does not require any ethical clearance from host institutions, we followed internationally accepted protocols [50]. This study was conducted in accordance with all relevant Indian laws, with due permits from the State Forest Departments of Kerala, Tamil Nadu and Karnataka as well as the Ministry of Environment and Forests, Government of India (Sanction Order No. 23/17/2003-RE).

### DNA extraction, amplification and sequencing

DNA was extracted using a DNeasy tissue kit (Qiagen, Hilden, Germany) following the manufacturer's protocols. We used three mitochondrial markers, cytochrome *b* (cyt *b*) 1067 base pairs (bp), cytochrome *c* oxidase subunit I (COI) 715 bp and Control Region (D-loop) 460 bp, and one nuclear marker, glyceraldehyde-3-phosphate dehydrogenase intron (G3PDH) 352 bp for this study. All the PCR reactions were carried out with 1X Taq Buffer B, 2 mM MgCl2, 1.5 U Taq DNA Polymerase (Bangalore Genei), 0.25 mM dNTP mix (Eppendorf, Hamburg, Germany), 0.2 µM of each primer (Sigma-Aldrich Chemicals, Bangalore, India), and about 1–1.5 µl of DNA extract.

We conducted a standard 35-cycle PCR with different primer sets (Table 3) to amplify all the target regions with a denaturation of 30 sec at 93–95°C, annealing for 30 sec at different temperatures for different markers and different sample locations. All PCR products were checked visually by running 2 µl of the product in 1% Agarose gels (Bangalore Genei, Bangalore, India) and were purified using Qiagen PCR purification kit (Qiagen, Hilden, Germany). The concentrations were measured by loading 2 µl of the PCR products in a spectrophotometer (NanoDrop ND-1000, Nanodrop Technologies, Delaware, USA). Forward and reverse sequences were obtained for all samples from Macrogen Inc. USA except for a few reactions of D-Loop (WBSDLPF for Peppara samples and Bababudan samples). The sequencing was conducted using an ABI 310 Genetic Analyzer and the raw sequences analysed with the ABI 310 Genetic Analyzer Version 3.1 software (Applied Biosystems, Foster City, USA).

**Table 3:** Markers, primers and annealing temperature for White-bellied Shortwing PCR reactions and outgroups used for phylogenetic analysis.

Marker	Primers	Annealing temperature	GenBank Accession
Cyt-b (1067 bp)	L14841 [73] and H15547 [74], H4A [75] and B5F [76]	66.7°C and 60°C North of Palghat Gap, 63°C South of Palghat Gap	GU644572 to GU644604
			Outgroups: EF081349, EU423183, AB353339
D-loop (460 bp)	primers in [77], WBSDLPF (5′-CTGGTTCCTATTTCAGGG-3′)	60°C North of Palghat Gap and 61.1°C South of Palghat Gap (with 50 cycles)	GU644605 to GU644637
			Outgroup: AY352167
Cox-1 (715 bp)	WBSCOXF (5′-TAAGCCTTCTCATCCGAG-3′) and WBSCOXR (5′-GGGCTCAGACGATAAAAC-3′)	51.1°C North of Palghat Gap and 50.9°C South of Palghat Gap	GU644539 to GU644571
			Outgroup: EF422246
G3PDH (352 bp)	Primers in [78]	67.3°C	GU644506 to GU644538
			Outgroup: FJ357918

Footnote: Marker codes: cyt *b* - cytochrome *b* , cox-1 - cytochrome *c* oxidase subunit I, D-loop - Control Region, G3PDH - glyceraldehyde-3-phosphate dehydrogenase intron.

### Analyses

The sequences were edited manually and assembled using the Mega 3.1 sequence editor [51]. They were multiple aligned using ClustalW [52] in Mega 3.1 Alignment Explorer with default parameters.

#### Biogeographic barriers and population structure

To understand population structure and to examine isolation by distance, average genetic distances between and within groups of broad geographical ranges were calculated using the Kimura-2-parameter model in Mega 3.1 [51]. For this analysis samples from Kodaikanal were grouped with samples from Grass Hills and referred to as Anamalai. This distance was calculated for each of the three mitochondrial regions (COI, cyt *b* and D-loop) separately, for the combined mitochondrial regions of COI and cyt *b* (denoted by MtDNA) and for the nuclear marker G3PDH. Pair-wise comparison of F_ST_ between populations of sampled Shortwings was also conducted with ARLEQUIN 3.11 [53] and significance values were determined with 1000 permutations in the same software. For this analysis too, the same population groups and the same marker sets as the previous analysis were used.

Population structure of the Shortwing was examined using phylogenetic analyses conducted using neighbour joining and maximum likelihood methods in PAUP* [54] and by using Bayesian inference in MrBayes Version 3.1 [55]. In all cases, trees were built independently for the different segments from cyt *b*, COI, D-loop, the combined mitochondrial DNA data of cyt *b* and COI (denoted as MtDNA), and for the segment of the G3PDH marker. An analysis of combined datasets of different markers was limited by the lack of data from an outgroup species. Wherever an outgroup species was available across markers, the combined analysis have been carried out. Prior to phylogenetic analyses, ModelTest Version 3.8 [56] was used to determine the evolutionary model that best fits the data based on Akaike Information Criterion for the separate and combined datasets. These analyses revealed that the evolutionary model that best fit the data in different regions was: GTR+I (General Time Reversible model, Tavare; [57]) for COI, TVM+G (Transversion model, Posada 2003; [58]) for cyt *b*, HKY+I+G (Hasegawa, Kishino and Yano model, Hasagewa; [59]) for D-loop, TVM+I (Transversion model, [58]) for combined MtDNA and HKY (Hasegawa, Kishino and Yano model, [59]) for G3PDH, where G stands for gamma distributed rate variation within sequence and I for proportion of invariable sites. The ML and NJ trees were reconstructed using the above evolutionary models and tested with 1000 bootstrap replicates each. The Bayesian Inference trees were reconstructed using MrBAYES v3.1.2 (Ronquist, 55) assuming only a GTR (for COI, cyt *b*, MtDNA markers) or HKY (D-loop and G3PDH markers) evolutionary model and by allowing the program to generate all the other parameters independently. Since the best-fit model for MtDNA and cyt *b*, TVM, is not available in MrBAYES, hence the next closest model GTR was used for analysis (as in Castoe [60]). The phylogenetic analyses were run until convergence with samples collected every 100 generations and 4 chains (1 cold and 3 heated) were used for the Markov Chain Monte Carlo (MCMC) procedure in all cases. The first 25% of the collected posterior data were discarded to allow ‘burnin’ [55]. A haplotype network was created with the cytochrome-b DNA (cyt *b*) data using the median joining (MJ) network algorithm [61] using NETWORK 4.5.0.2 (fluxus-engineering.com).

Population structure of the Shortwing was also examined using Analysis of variance (AMOVA) computed using ARLEQUIN 3.11 [53]. AMOVA as implemented in ARLEQUIN allows us to (1) describe the partitioning of genetic variation among and within groups, and (2) investigate the contributions of different user-defined population grouping scenarios to the partitioning of genetic variation in the dataset [53]. We explored various models of partitioning of genetic variation (Supplementary Table S1). The grouping that showed highest partitioning of variation among groups and the lowest among and within populations was selected as the best model of genetic structure.

To test for isolation by distance, we conducted a Mantel test [62] we checked for correlation between genetic distance (pair-wise F_ST_) matrix and the geographical distance matrix and also the effect of Gaps by using a third dummy variable indicating the locations of the Gaps using the methods described in Bohonak 2002 [63] and implemented in IBDWS (ver 3.16) [Jansen 64]. Using these methods we were able to examine the correlation between any two variables in addition to which, we also conduct a partial mantels test where partial correlations provided us the significance of correlation of two variables while controlling for the effect of the third variable. The geographic distance was calculated in kilometres from GPS locations plotted on the Google Earth database (http://earth.google.com). The correlation was tested for statistical significance at 0.05 level using a randomization test with 1000 iterations [65].

#### Evolutionary history and impacts of climate

To investigate the evolutionary history of the Shortwing, we estimated dates of divergence between and within clades with cyt *b* data using BEAST, version 1.4.8 [66] (0.0105 substitutions per lineage per million year (Myr), strict molecular clock, GTR+I model of substitution, Yule process tree prior following recommendation of Drummond *et al.* [67]. The widely used sequence divergence rate of 2.1% Myr^−1^ for cyt *b* has been validated by a recent study by Weir and Schluter [68] who found 2.1% Myr^−1^±0.1% (95% confidence interval) across 12 taxonomic orders. Hence we used this divergence rate for estimating divergence times across Shortwing clades. Range expansion events have been dated by calculating Tau = 2 µt [69] based on mismatch distributions generated by ARLEQUIN [53]. We used the mutation rate of 2.1% Myr^−1^ and generation time of 1 year for this calculation. The differences between the distribution of pairwise differences in a set of sequences to a distribution based on a population expansion model or ‘mismatch distribution’ [70] has often been used to infer demographic history [71], [72]. We examined mismatch distributions based on a population expansion model in a maximum likelihood framework using ARLEQUIN [53] with 1000 bootstrap iterations.

## Supporting Information

Figure S1 Phylogenetic tree (Bayesian) of White-bellied Shortwing on sky-islands of southern India based on 998 bp of the cytochrome-b marker. Branch support values indicate Bayesian, Neighbour-Joining bootstrap support and Maximum Likelihood bootstrap support and values. The samples from a region are coloured by ‘island’ colour in the map.
(5.65 MB TIF)

Figure S2 Phylogenetic tree (Bayesian) of White-bellied Shortwing on sky-islands of southern India based on 714 bp of the cytochrome oxidase-1 marker. Branch support values indicate Bayesian, Neighbour-Joining bootstrap support and Maximum Likelihood bootstrap support and values. The samples from a region are coloured by ‘island’ colour in the map.
(3.74 MB TIF)

Figure S3 Phylogenetic tree (Bayesian) of White-bellied Shortwing on sky-islands of southern India based on 461 bp of the Control Region marker. Branch support values indicate Bayesian, Neighbour-Joining bootstrap support and Maximum Likelihood bootstrap support and values. The samples from a region are coloured by ‘island’ colour in the map.
(4.78 MB TIF)

Figure S4 Phylogenetic tree (Bayesian) of White-bellied Shortwing on sky-islands of southern India based on 352 bp of the nuclear glyceraldehyde-3-phosphate dehydrogenase intron (G3PDH) marker. Branch support values indicate Bayesian, Neighbour-Joining bootstrap support and Maximum Likelihood bootstrap support and values. The samples from a region are coloured by ‘island’ colour in the map.
(4.70 MB TIF)

Table S1 Partitioning of genetic variation of White-bellied Shortwing populations in the Western Ghats sky islands based on mitochondrial DNA (2186 bp, COI + Cyt b + D-loop) Footnote:*Significant (p<0.05), + Population codes: 1-Bababudan, 2- Ooty, 3- Grasshills, 4-Kodiakanal, 5- High-Wavies, 6- Peppara; Brackets indicate grouping of population. Groupings that include populations on either side of the gaps (e.g. (1,2,5), 3, 4, 6; (1,2,3), 4, 5, 6; 1, (2, 3), 4, 5, 6) result in negative among group variance, and hence are not included in this table.
(0.05 MB DOC)
