# Supplementary material for: Ancient Geographical Gaps and Paleo-Climate Shape the Phylogeography of an Endemic Bird in the Sky Islands of Southern India
Source: PLoS One. 2010 Oct 13;5(10):e13321. doi: 10.1371/journal.pone.0013321 (PMC2954160; doi:10.1371/journal.pone.0013321)
Supplement: Table S1 — Partitioning of genetic variation of White-bellied Shortwing populations in the Western Ghats sky islands based on mitochondrial DNA (2186 bp, COI + Cyt b + D-loop) Footnote:*Significant (p<0.05), + Population codes: 1-Bababudan, 2- Ooty, 3- Grasshills, 4-Kodiakanal, 5- High-Wavies, 6- Peppara; Brackets indicate grouping of population. Groupings that include populations on either side of the gaps (e.g. (1,2,5), 3, 4, 6; (1,2,3), 4, 5, 6; 1, (2, 3), 4, 5, 6) result in negative among group variance, and hence are not included in this table. (0.05 MB DOC) [file pone.0013321.s005.doc]

Supplementary Table (ST1): Partitioning of genetic variation of White-bellied Shortwing populations in the Western Ghats sky islands based on mitochondrial DNA (2186bp, COI + Cyt *b* + D-loop)

| Population grouping Model | Among Group Variation | Among Population Within Group Variation | Within Population Variation | F_ST_ |
| --- | --- | --- | --- | --- |
| (1,2), 3, 4, 5, 6 | 85.3 | 6.48 | 7.71 | 0.923* |
| 1,2, (3, 4, 5, 6) | 77.17 | 19.70 | 3.12 | 0.969* |
| 1, (2, 3, 4, 5, 6) | 62.58 | 34.13 | 3.2 | 0.967* |
| (1, 2), (3, 4, 5, 6) | 79.29 | 17.73 | 2.97 | 0.97* |
| (1, 2), (3, 4, 5), 6 | 93.15 | 1.15 | 5.70 | 0.943* |

*Significant (p<0.05), ^+^ Population codes: 1-Bababudan, 2- Ooty, 3- Grasshills, 4-Kodiakanal, 5– High-Wavies, 6– Peppara; Brackets indicate grouping of population. Groupings that include populations on either side of the gaps (e.g. (1,2,5), 3, 4, 6; (1,2,3), 4, 5, 6; 1, (2,3), 4, 5, 6) result in negative among group variance, and hence are not included in this table
